# Chromium released from leather – II: the importance of environmental parameters

**DOI:** 10.1111/cod.12334

**Published:** 2015-01-29

**Authors:** Frederik Mathiason, Carola Lidén, Yolanda S. Hedberg

**Affiliations:** ^1^ Division of Surface and Corrosion Science, Department of ChemistrySchool of Chemical Science and Engineering, KTH Royal Institute of Technology, Drottning Kristinas väg 51SE‐10044StockholmSweden; ^2^ Unit of Occupational and Environmental DermatologyInstitute of Environmental Medicine, Karolinska Institutet, Nobels väg 13Box 210SE‐17177StockholmSweden

**Keywords:** allergic contact dermatitis, chromium(III), chromium(VI), environment, humidity, leather, metals, occupational

## Abstract

**Background:**

Approximately 1–3% of the adult population in Europe are allergic to chromium (Cr). A new restriction in Registration, Evaluation, Authorization and Restriction of Chemicals (REACH) based on the ISO 17075 standard has recently been adopted in the EU to limit Cr(VI) in consumer and occupational leather products to < 3 mg/kg.

**Objectives:**

To investigate the influence of storage conditions [relative humidity, temperature, ultraviolet (UV) irradiation, and duration] on Cr release, and to assess several parameters relevant for occupational exposure (repeated exposure, wear, alkaline solutions, and sequential wet and dry exposures).

**Material and methods:**

A leather of relevance for work gloves was investigated for its release of Cr(III) and Cr(VI) under these different experimental conditions.

**Results:**

Relative humidity (water content in leather) during storage prior to Cr extraction was the single most important parameter. Cr(VI) levels could vary from non‐detectable to levels significantly exceeding the restriction limit, depending on the relative humidity. Leather contact with alkaline solution and UV irradiation during storage could increase the Cr(VI) levels in subsequent extractions.

**Conclusions:**

The amount of Cr(VI) in leather is not an intrinsic property, but is influenced by environmental conditions of relevance for occupations and skin exposure.

Contact allergy to chromium (Cr) is the third most common metal allergy, after allergy to nickel and cobalt, affecting approximately 1–3% of the adult general population 1. Since the 1990s, leather products have attracted increasing attention as a cause of Cr allergy and dermatitis 2, 3. Between 7% and 50% of ∼ 9500 leather products tested and reported since the year 2000 contained Cr(VI) at concentrations above the limit of detection (3 mg/kg_leather_) of the ISO 17075 standard 4, 5, 6, 7, 8. A limitation of Cr(VI) in leather was initially proposed by Denmark 9, and it is anticipated that a restriction will enter into force within EU in 2015 10. It is based on the ISO 17075 standard 11 for leather products, which stipulates Cr(VI) determination by extraction of leather powder in de‐aerated phosphate buffer for 3 hr.

We have previously quantified the release of trivalent Cr [Cr(III)] and hexavalent Cr [Cr(VI)] from differently tanned, unfinished and thoroughly characterized leather samples 12. We have also investigated the influence of stipulated test conditions of the ISO 17075 standard, and other key exposure parameters, such as temperature, duration, surface area, and solution de‐aeration 13. It was found that significantly more Cr(III) than Cr(VI) was released under all conditions and for all investigated leather samples. Several test conditions were proposed to influence the amount of extracted Cr(VI): grinding or cutting of the sample, the extraction temperature (not defined in the standard), and reductive species (antioxidants) in the leather. The storage conditions prior to extraction measurement with the ISO 17075 standard are undefined, but might be of great importance for the extraction result and also occupational exposure.

The objectives of this study were to investigate the influence of storage conditions [relative humidity (RH), temperature, ultraviolet (UV) irradiation, and duration] prior to extraction, and several parameters relevant for occupational exposure (repeated exposure, wear, alkaline solutions, and sequential wet and dry exposures), for leather of relevance for work gloves.

## Materials and Methods

### Leather used in this study

The leather (from cattle; one large sample of size ∼ 0.5 m^2^) used throughout this study was from normal production and was received from a European tannery. All pieces in this study (sized 1.0 × 1.0 × 0.1 cm; triplicate samples for each tested condition) were cut from the large sample of leather. The leather was Cr‐tanned and Cr‐post‐tanned, not coated, and without finish (so‐called crust leather). It was intended for use in work gloves (generally low‐price leather), thus representing leather available on the European market of relevance for human and environmental exposure. This leather was the same leather sample as denoted Cr^Cr^_gloves_ in 12, 13. Non‐coated samples were chosen to enable comparison between Cr release and Cr oxidation state on the leather surface. The leather was characterized in 12.

One sample of leather from a purchased work glove (Berner, art. no. 34034, size 9, pig leather) was also tested. The glove appeared to be unused and clean.

### Pretreatment and conditioning

Triplicate leather samples (approximately 1.0 × 1.0 × 0.1 cm, from the large cattle leather described above) were exposed in parallel to each test condition. Different exposure conditions of relevance for occupational scenarios and environments were chosen. The effects of RH, temperature, storage time and UV irradiation (sunlight) were investigated to simulate different scenarios of air exposure of leather. It has previously been shown that exposure to air under certain conditions can increase the extent of Cr(VI) released 13, 14. Cr(III) and Cr(VI) release was investigated in artificial rain, alkaline solution [to simulate, for example, excess water in cement work 15], and phosphate buffer (PB) 11. All effects were investigated individually and in different combinations, mimicking a normal workday, during which the work glove might be exposed to rain, storage, and sunlight. To control the RH and temperature, an environmental chamber (Weiss WK3‐340/40) was used. The UV irradiation emanated from a UV‐light source of 15 W, placed 25 cm from the sample, which was irradiated for 4 hr (long enough to ensure drying and any oxidation). To investigate the effect of wear (stretching) on Cr release from leather, one leather sample (6.9 × 2.4 cm) was stretched with a tensile test machine (Instron 5566 Universal Testing Machine), for 1000 cycles (each 20 seconds) with 15% strain, in total for 5 hr. In a pre‐study, these conditions were found to be optimal for plastic (irreversible) stretching of this leather sample. After the stretching, the leather sample had no visible ruptures, but had changes in fibre structures that were visible with the naked eye. This leather sample was then cut into three samples and exposed to storage at 20% RH and 70°C for 24 hr, with subsequent extraction in PB for 3 hr.

### Extraction

The artificial rain was based on 16 and contained 1.17 mg/l S (SO_4_^2−^), 0.36 mg/l Cl^−^, 0.56 mg/l N (NO_3_^−^), 0.56 mg/l N (NH_4_^+^), 0.23 mg/l Na^+^, 0.12 mg/l K^+^, 0.12 mg/l Mg^2+^, and 0.20 mg/l Ca^2+^ (pH 4.3), prepared by mixing NH_4_NO_3_, Na_2_SO_4_, K_2_SO_4_, MgSO_4_.7H_2_O, CaCl_2_.2H_2_O, and H_2_SO_4_, all analytical grade, with ultrapure water (18.2 MΩ cm; Millipore, Solna, Sweden). The alkaline solution was composed of 7.85 g/l Na_2_HPO_4_ and 1.4 g/l NaOH (pH 12.2). The PB was composed of 11.8 g/l K_2_HPO_4_.3H_2_O, adjusted to pH 8.0 ± 0.1 with 70 vol.% phosphoric acid, and de‐aerated prior to the extraction test, according to 11. The phosphate concentration of 11.8 g/L in this study is lower compared to 22.8 g/L in 11, but this difference was found to be of minor importance, as extracted total Cr and Cr(VI) from the cattle leather (stored for 24 h at 70°C and 20% RH) for 3 hr in these two solutions only differed 0–20% (data not shown). Ultrapure water (18.2 MΩ cm) was used as the solvent for all solutions, and all equipment was acid‐cleaned prior to use (10% HNO_3_ for at least 24 hr), and rinsed four times with ultrapure water. Extraction was conducted in PB (pH 8.0) for 3 hr, according to 11, and in artificial rain (for 3 and 6 hr) and alkaline solution for 3 hr. The extraction was performed at room temperature (20–25°C) with bilinear shaking (22 cycles/min, 12°C), in 5 ml of solution (∼ 50 mg of leather sample in 5 ml of solution). After extraction, the solution was centrifuged (704 g) to remove any released leather fibres, and frozen prior to Cr(VI) analysis, or acidified (pH < 2) prior to total Cr analysis by atomic absorption spectroscopy (AAS).

### Atomic absorption spectroscopy

The total amount of Cr released was determined by the use of AAS with calibration standards of 0, 0.5, 1.5, 5 and 10 mg/l Cr (in 1% HNO_3_). The limit of determination was estimated to 0.018 mg Cr/l, as determined by the highest standard deviation of the measured blanks, multiplied by 3. The concentrations of all blanks were below the instrument limit of detection, that is, showing no positive values, and were therefore accounted as zero. After four samples, quality controls of known concentrations were measured. If the measured control sample deviated by > 10%, recalibration was performed.

### Spectrophotometry and diphenylcarbazide (DPC)

To determine the amount of Cr(VI) in the extract (frozen prior to analysis), spectrophotometry was performed, utilizing the pink colour of the complex between Cr(VI) and DPC 17, with an absorption maximum at 540 nm. In accordance with the standard test 11, all samples, phosphoric acid (70 vol.%) and DPC solution (1.0 g DPC in 100 ml of acetone acidified with one drop of glacial acetic acid) were mixed in the ratio of 96 vol.% sample, 2 vol.% phosphoric acid, and 2 vol.% DPC solution. Calibration standards were prepared of concentrations 0, 125, 247.5, 495 and 990 µg Cr(VI)/l in the solution investigated (i.e. different calibration curves for different solutions). The limit of determination was found for each calibration curve [38–48 µg Cr(VI)/l] by multiplying the highest standard deviation of the blank values by 3, and the calibration curves were linear (*R* ^2^ = 0.9949–0.9996). All measured blank values were below the limit of determination, and did not differ by > 0.02 absorbance from zero (lower than the limit of determination).

**Figure 1:**
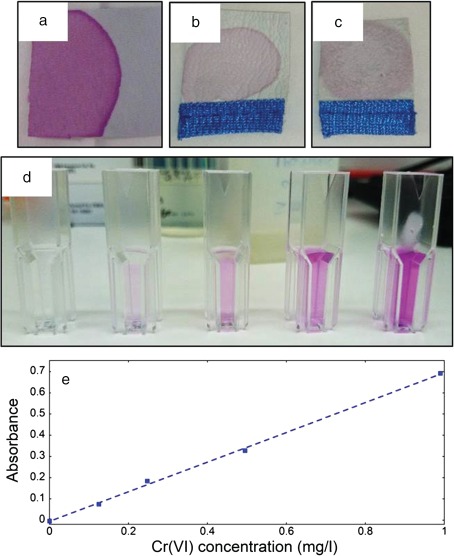
Diphenylcarbazide (DPC) solution directly applied to leather samples: Cr‐tanned and Cr‐post‐tanned leather (cattle) (a); purchased work glove (pig) inner side (b) and outer side (c); DPC colouring the Cr(VI) calibration solution standards (d) used for the calibration curves [example in (e)].

The calibration standards and a calibration curve are shown in Fig. 1, together with the DPC solution directly applied to both leather samples. DPC solution directly applied to dry leather samples can indicate whether Cr(VI) is present in the sample 12, 18, but artefacts are theoretically possible when the leather is in contact with air, owing to the oxidation of DPC to diphenylcarbazone at neutral or alkaline pH and subsequent complexation with Cr(III) 17.

### Presentation of data

**Figure 2:**
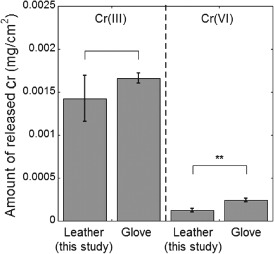
The amounts of Cr(III) and Cr(VI) (mg/cm^2^) released in phosphate buffer (PB) after 3 hr of extraction at room temperature (20–25°C) for two different types of leather: leather (this study, cattle) and work glove (pig). Prior to PB extraction, the leather was stored and conditioned at 70°C and relative humidity 20% for 24 hr. The error bars indicate the standard deviation between triplicate samples. The asterisks represent significant differences between the leather types (*p < 0.05; **p < 0.01; no asterisk, p ≥ 0.05). Corresponding values in mg/kg are given in Table S1 of Appendix S1.

**Figure 3:**
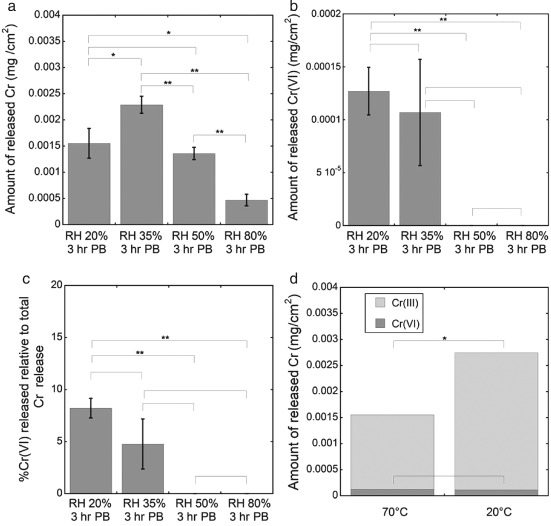
The amount of Cr released in phosphate buffer (PB) after 3 hr of extraction at room temperature (20–25°C). (a–c) Prior to PB extraction, the leather (cattle) was stored and conditioned at different relative humidities (RHs) for 24 hr. In (c), the same data as in (b) are shown as percentage Cr(VI) of total Cr released. (d) Cr release after pre‐storage at 20% RH for 24 hr at different temperatures. The error bars indicate the standard deviation between triplicate samples. The asterisks represent significant differences (*p < 0.05; **p < 0.01; no asterisk, p ≥ 0.05). Corresponding values in mg/kg are given in Table S1 of Appendix S1.

**Figure 4:**
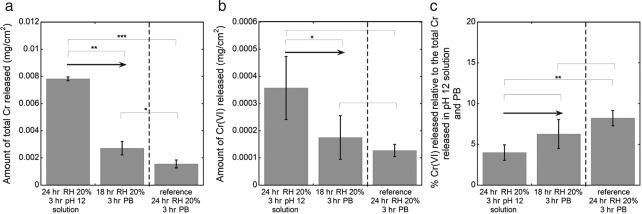
The amounts of total Cr released (a), Cr(VI) released (b) and Cr(VI) released as a percentage of the total release (c) in alkaline solution (pH 12) and phosphate buffer (PB). The leather (cattle) was stored and conditioned at 70°C and 20% relative humidity (RH) for 24 hr, extracted in alkaline solution for 3 hr, dried at room temperature (20–25°C) and 20% RH for 18 hr, and finally extracted in PB for 3 hr at room temperature (20–25°C). A reference (stored/conditioned at 70°C and 20% RH for 24 hr, and then extracted in PB for 3 hr) is shown for comparison. The error bars indicate the standard deviation between triplicate samples. The asterisks represent significance (*p < 0.05; **p < 0.01; ***p < 0.0001; no asterisk, p ≥ 0.05). Corresponding values in mg/kg are given in Table S1 of Appendix S1.

**Figure 5:**
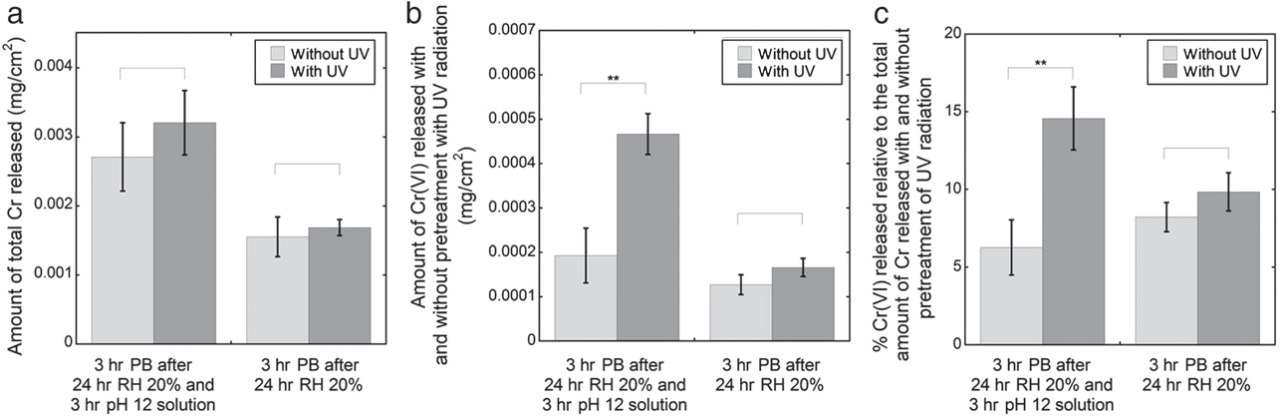
The amounts of total Cr released (a), Cr(VI) released (b) and Cr(VI) released as a percentage of the total release (c) in phosphate buffer (PB) after 3 hr of extraction at room temperature (20–25°C). Left bars: the leather (cattle) was first stored and conditioned at 70°C and 20% relative humidity (RH) for 24 hr, extracted in alkaline solution for 3 hr, dried at room temperature (20–25°C) and 20% RH for 18 hr, irradiated or not irradiated with ultraviolet (UV) radiation for 4 hr at 20% RH at room temperature (20–25°C), and finally extracted in PB for 3 hr at room temperature. The right bars represent a reference test in which the leather (cattle) was stored and conditioned at 70°C and 20% RH for 24 hr, irradiated or not irradiated with UV radiation for 4 hr at 20% RH and 20–25°C, and then extracted in PB for 3 hr. The error bars indicate the standard deviation between triplicate samples. The asterisks represent significance (*p < 0.05; **p < 0.01; no asterisk, p ≥ 0.05). Corresponding values in mg/kg are given in Table S1 of Appendix S1.

**Figure 6:**
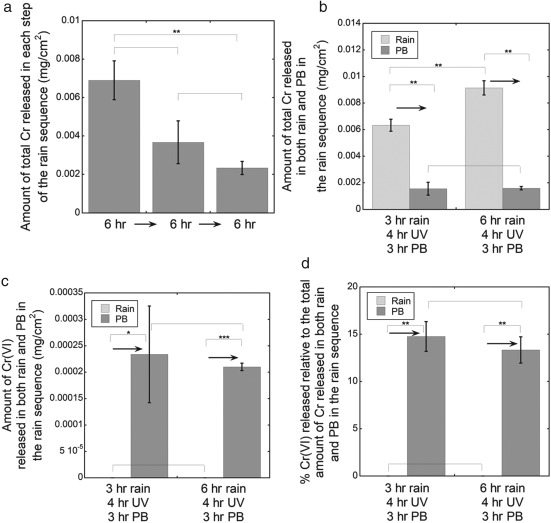
The total amount of Cr released from the leather (cattle) in each step of the repeated extraction in artificial rain (rain, pH 4.3) (a), the total amount of Cr released in either rain or phosphate buffer (PB) extracted first in rain and then in PB [after ultraviolet (UV) irradiation] (b), the amount of Cr(VI) released in either rain or PB extracted first in rain and then in PB (after UV irradiation) (c), and Cr(VI) released as a percentage of the total release, extracted first in rain and then in PB (after UV irradiation) (d). Storage conditions: 18 hr at room temperature (20–25°C) and 20% relative humidity (RH) between the rain extractions (a), and 20–23 hr at room temperature (20–25°C) and 20% RH prior to UV irradiation for 4 hr (20–25°C, 20% RH) and storage for 19 hr (20–25°C) (b–d). The error bars indicate the standard deviation between triplicate samples. The asterisks represent significance (*p < 0.05; **p < 0.01; no asterisk, p ≥ 0.05). Corresponding values in mg/kg are given in Table S1 of Appendix S1.

**Figure 7:**
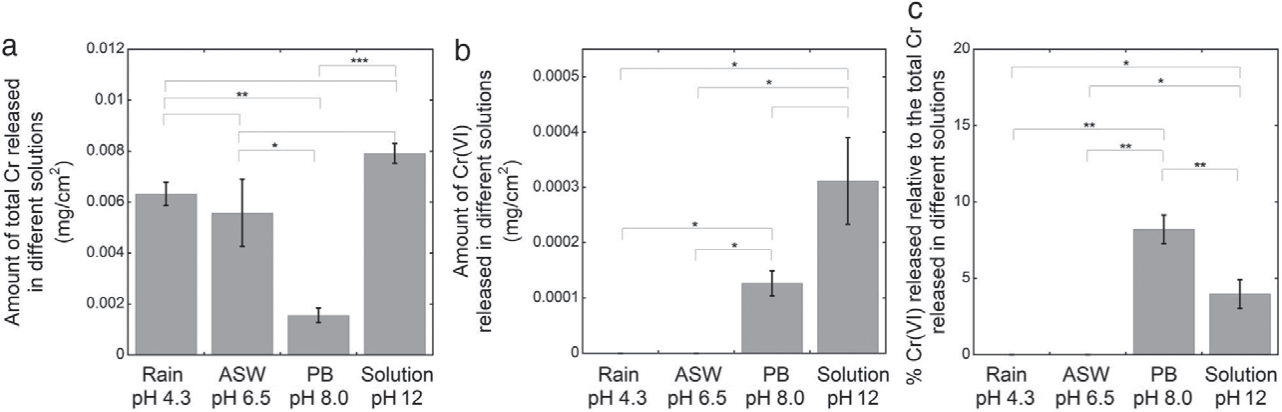
The amounts of total Cr released (a), Cr(VI) released (b) and Cr(VI) released as a percentage of total release (c) in artificial rain (rain, pH 4.3), artificial sweat (ASW, pH 6.5), phosphate buffer (PB, pH 8.0) and alkaline solution (pH 12) for 3 hr (each). Data for ASW were taken from 12. The error bars indicate the standard deviation between triplicate samples. The asterisks represent significance (*p < 0.05; **p < 0.01; ***p < 0.0001; no asterisk, p ≥ 0.05). The non‐significant difference in (b) had a p‐value of 0.07. Corresponding values in mg/kg are given in Table S1 of Appendix S1.

**Figure 8:**
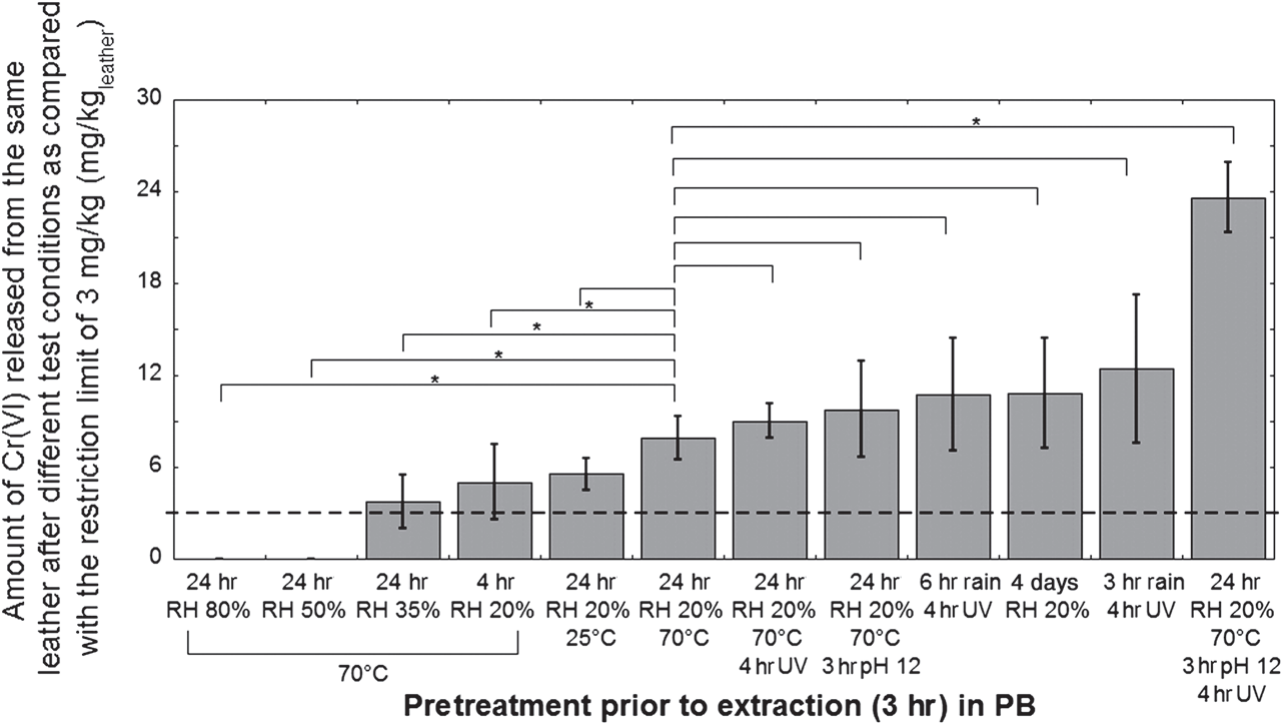
Amount of Cr(VI) released from the same type of leather (cattle), normalized over mass, in phosphate buffer (PB) after extraction for 3 hr at room temperature (20–25°C) under different environmental conditions prior to extraction. The dotted line indicates the restriction limit (3 mg/kg). The asterisks represent significance (*p < 0.05; no asterisk, p ≥ 0.05) with respect to the sample pretreated for 24 hr at 70°C and 20% relative humidity (RH) after extraction for 3 hr at room temperature (20–25°C). UV, ultraviolet.

The data are presented as mean values with error bars indicating standard deviations of triplicate samples. The majority of the data are normalized to the surface area (mg/cm^2^); this has been suggested to be the most relevant way of presenting Cr release from leather in earlier studies 13. In Fig. 8, the Cr(VI) release is compared with the EU restriction limit of 3 mg/kg [mg Cr(VI) per kg of dry leather] 19, and is therefore presented in mg/kg. The surface area and mass of each sample were measured before exposure. For comparison, corresponding values in mg/kg for Figs. 2, 3, 4, 5, 6, 7 and Fig. S1 are given in Table S1 of Appendix S1. Statistical significance was evaluated with Student's *t*‐test (two‐sided) with unequal variance for unpaired data, or for paired data (when the same samples were exposed in a sequence; shown as arrows in figures). The word ‘significant’ in the text refers to a *p*‐value of < 0.05.

## Results

### Comparison of leather samples

The release of Cr(III) and Cr(VI) from the leather used throughout this study (from cattle) was compared with release from a bought leather work glove (from pig). It was found that the amount of Cr(III) released did not differ significantly between the two leathers. However, the release of Cr(VI) differed significantly, with significantly more Cr(VI) being released from the working glove (Fig. 2). Both leathers became pink when a drop of DPC solution was applied to the dry leather (Fig. 1), indicative of Cr(VI) being present in the leather 12, 18. The amount of Cr(VI) released from both leathers (5–19 mg/kg) is within the values reported in the literature (< 1–96 mg/kg_leather_) 4, 5, 6, 7, 20. In the following, all results presented refer to the leather from cattle.

### Effect of RH and temperature during storage

Figure 3 shows that the RH significantly affected the total amount of Cr released from the leather. There was a decreasing trend in Cr(VI) release with increasing RH (not significant, between 20% and 35% RH), until the RH reached a threshold value above which no detectable Cr(VI) was released. From Fig. 3d, it is also evident that temperature affected the total amount of Cr released, with more Cr being released after storage at lower temperature and 20% RH. Together, these observations show that the moistness (water content) of the leather strongly affects Cr(VI) release: less Cr(VI) is released with increasing leather water content during storage.

### Contact with alkaline solution

Contact with alkaline solution of pH 12 significantly increased the total amount of Cr released from the leather in both the alkaline solution and with subsequent (indicated by arrows) extraction in PB, as shown in Fig. 4a. The total amount of Cr in the alkaline solution was higher than with subsequent extraction in PB, and also higher than with direct extraction in PB (without pre‐extraction), used as a reference. This means that a pre‐extraction in alkaline solution of pH 12 [relevant, for example, in work with excess water that has been in contact with cement 15] can increase Cr release in subsequent extractions.

### UV irradiation during storage in air

Although the total amount of Cr released was unaffected by 4 hr of UV irradiation at 20% RH and 20–25°C prior to extraction, the amount of Cr(VI) released was affected (Fig. 5). This trend was significant when the leather had been extracted in the alkaline solution (3 hr) prior to storage (20–25°C, 20% RH, 18 hr) and subjected to 4 hr of UV irradiation (Fig. 5b,c).

### Effect of repeated usage and wear

As the leather is used, Cr is released (leached out), as seen in Fig. 6a for repeated (indicated by arrows) extraction in artificial rain. A decrease in the total amount of Cr released from the leather upon repeated extraction was found. No significant difference was observed between the steps (6 hr of extraction), other than between the first and the last step. No Cr(VI) was released in artificial rain (pH 4.3). From Fig. 6b, it is evident that the amount of Cr released from the leather in the subsequent PB extraction remained the same, despite the pre‐extraction in artificial rain. Whereas no Cr(VI) was released in the artificial rain, it was released from the same leather in the subsequent extraction in PB after 20–23 hr of storage at 20% RH and 20–25°C, and after 4 hr of UV irradiation at 20% RH and 20–25°C. The artificial wearing out of the leather by stretching did not have any significant effect on total Cr or Cr(VI) release after extraction in PB for 3 hr (Fig. S1 of Appendix S1).

### Effect of the pH and composition of the extraction solution

The pH affected the release of both total Cr and Cr(VI), as seen in Fig. 7. No significant difference in total Cr released between extraction in artificial rain (pH 4.3), artificial sweat (ASW, pH 6.5) or alkaline solution (pH 12) was observed, but all of these solutions extracted significantly higher amounts of total Cr than PB (pH 8.0). Cr(VI) was only released in alkaline solutions (Fig. 7b), and the amount released increased with increasing pH. In PB, the highest percentage of Cr(VI) as compared with total Cr released was observed (Fig. 7c).

### Comparison with the future restriction limit of 3 mg/kg Cr(VI)

Figure 8 shows Cr(VI) extracted, according to the test protocol 11, from the same type of leather but with different pretreatments prior to extraction. Storage, preconditioning and the history of the leather samples to be tested are not defined in the test protocol. The results are presented in mg/kg, and 3 mg/kg is the restriction limit. It is evident that the same leather, depending on pretreatment and conditions, can give a negative test result, a positive test result, or a very positive test result. For the leather tested, only a few conditions (50 and 80% RH) would result in concentrations below the restriction limit. It should be underlined that the RH is the single most important parameter affecting the outcome of the test for Cr(VI) in leather, followed by exposure to UV (relevant for storage in sunlight), in combination with different pretreatments under dry conditions, and contact with alkaline media (Fig. 8).

## Discussion

In this study, we focused on leather of relevance for work gloves. This leather is sometimes coloured, but is often unfinished (not coated/sprayed), owing to the expected wear (damaged coatings) during use. There are many different Cr‐tanned leather work gloves on the market, intended for different tasks and occupations. Different gloves on the market undergo different tanning processes, and they are exposed to different environments. The results of this study are therefore not fully applicable to all of these gloves and occupations. However, general conclusions on the effects of different parameters can be drawn from this study, and are considered to be relevant for all leather work gloves, unless they contain large amounts of added antioxidants hindering the release of Cr(VI) 20, 21, 22, 23, 24, 25.

In accordance with the literature 14, 20, 24, 26, it was evident that the water content (moistness) of the leather was the single most important parameter influencing Cr(VI) release. With increasing water content in the leather during pre‐storage, the amount of Cr(VI) extracted decreased, until it was not detectable at an RH of > 35% at 70°C. Several mechanisms could contribute to this strong trend. (i) The water could shield any free Cr(III) from being oxidized by oxygen from the air. (ii) The water could mobilize any reducing agents (antioxidants) present in the leather, such as vegetable tannins, added antioxidants, or different acids (sulfuric acid, oxalic acid, or formic acid) 27, which would reduce any Cr(VI) or hinder oxidation from Cr(III) to Cr(VI), for example by decreasing the pH. The strong influence of the leather water content, determined by the temperature and RH during storage prior to the extraction test, is most important, as this parameter is not defined in the test protocol 11, and varies substantially among countries. The condition under which a negative test result for Cr(VI) was found in this study, an RH of > 35% prior to testing, occurs frequently in many countries, where the temperature outside of the laboratory is equal to or warmer than inside, and/or where high humidity is usual. The relative humidity decreases in heated air, unless the air is humidified. It is therefore more probable that a sample will test positive in a dry and cold country than in a warmer or more humid country. Workers in countries where the climate is dry and cool may therefore be subjected to higher release of Cr(III) and/or Cr(VI) from the same gloves than workers in countries where the average temperature is similar to or higher than the indoor temperature, unless the RH is controlled (>35%) during storage. Control of the RH to ∼ 20% for at least 24 hr prior to the extraction test with the standard protocol 11 is therefore highly recommended.

In many occupations, alkaline solutions, such as water or sweat in contact with concrete or cement (dry or wet), are common, with pH values of 10–12 or higher 15. At this high pH, Cr(VI) is theoretically more stable and Cr(III) more unstable than at lower pH values 28. Also, above a pH of 8.3 [isoelectric point of collagen 29], leather swells (retains water) 30 and is negatively charged, which might change the Cr speciation and Cr release. In this study, we showed that a phosphate‐buffered alkaline solution (pH 12.3) increased total Cr release as compared with PB (pH 8.0), but that the percentage of Cr(VI) released was lower at pH 12. We speculate that this is mostly related to the swelling of the leather and change in collagen charge, possibly resulting in more Cr(III) being released (that has been bound to collagen). Importantly, Cr(III) release after contact with an alkaline solution is also increased, and the formation of Cr(VI) after contact with alkaline solution seems to be facilitated, as is evident from a significant increase in Cr(VI) release upon UV irradiation (not seen for the reference sample without previous contact with alkaline solution).

The higher Cr(III) release in artificial rain (pH 4.3) and ASW (pH 6.5) than in PB (pH 8.0) might be related to the lower pH [higher solubility of Cr(III) 28, 31] and higher NaCl content in the case of ASW 32. However, the trend may be different for other leathers, as a combination of leather and chromium chemistry (including the influence of vegetable tannins, colours, and coatings) determines the dependence on solution composition and pH, as is evident for differently tested leather samples in a previous study 13.

In a previous study on the effect of UV irradiation, no significant influence was found under wet conditions with similar test conditions and for the same leather as in this study 13. In other studies, mainly investigating UV irradiation (under dry conditions) used as a drying procedure after tanning, a significant increase in Cr(VI) release was found 20, 21, 22, 23, 24, 33, 34, 35. In the present study, we investigated UV irradiation of relevance for storage in the sun [4 hr of irradiation at low temperature (20–25°C)] prior to extraction. A significant effect was found in some cases for Cr(VI) as compared with references. The results suggest that UV irradiation during storage is not an important parameter itself, but may be of importance when combined with other conditions, for example contact with alkaline solutions prior to UV irradiation.

In accordance with previous studies in PB for the same leather 13, repeated extraction in artificial rain generally resulted in less Cr(III) being released for each step. However, after exposure to UV irradiation or alkaline solution (changes in collagen structure), subsequent extractions resulted in elevated amounts of Cr(III) and Cr(VI) being released as compared with reference tests. A previous Cr extraction in artificial rain, in which no Cr(VI) is released, does not, for example, protect against the formation and release of Cr(VI) in a subsequent extraction in PB. The storage conditions are of higher importance for the formation and release of Cr(VI) than any previous release. Therefore, Cr(VI) release cannot be an intrinsic property of leather, being determined only by the material, and not the environment.

In accordance with previous studies 6, 12, 13, 23, 36, 37, significantly more Cr(III) than Cr(VI) was released under all studied conditions. However, recent studies have highlighted the significance of Cr(III) for Cr contact dermatitis. It was shown by patch testing that Cr(III) concentrations as low as 0.18 µg/cm^2^ could elicit eczema in 10% of Cr‐allergic persons [minimum elicitation threshold (MET)_10%_] 38. This is only six times as high as the threshold value found for Cr(VI) (MET_10%_ 0.03 µg/cm^2^) 38. In this and previous studies on the same leather and other leathers, Cr(III) was released up to 72 µg/cm^2^ leather sample (168 hr in ASW), and Cr(VI) up to 0.56 µg/cm^2^ (3 hr in PB). Although these figures cannot be directly compared, they emphasize that the released amounts of both Cr(III) and Cr(VI) may pose a hazard.

## Conclusions and Future Perspectives

1. Significantly more Cr(III) than Cr(VI) was released under all tested conditions. Cr(VI) release from leather is not an intrinsic property of the leather material, but is influenced by environmental conditions of relevance for occupations and skin exposure.
2. It was found that the water content of leather in contact with air is the single most important parameter for total Cr and Cr(VI) release from leather.
3. Contact with alkaline solution resulted in increased amounts of both total Cr and Cr(VI) release, and could trigger the formation of Cr(VI) by UV irradiation.
4. UV irradiation during storage in air results in increased amounts of Cr(VI) release under some conditions.
5. Repeated usage results in repeated Cr release in generally decreasing amounts. However, the environmental conditions prior to extraction are more important than the history or wear of the gloves.
6. The pH and composition of the extraction solution strongly influenced total Cr and Cr(VI) release.
7. The test protocol ISO 17075 for the recently adopted restriction on Cr(VI) released from leather in the EU does not consider the environmental conditions prior to extraction, which were shown to strongly influence the outcome of the test. It is therefore highly recommended to store the samples at 20% RH prior to extraction.
8. Further studies should investigate the amount of Cr actually deposited on the skin during contact with Cr‐tanned leather, and correlate the amounts of different Cr species on the skin. Furthermore, it should be investigated whether environmental conditions are of substantial importance for total and Cr(VI) release after exposure of leather for more than a period of weeks.

## Supporting information


**Appendix S1.** Chromium released from leather – II: the importance of environmental parameters.
