# Supplementary material for: Chromium released from leather – II: the importance of environmental parameters
Source: Contact Dermatitis. 2015 Jan 29;72(5):275–85. doi: 10.1111/cod.12334 (PMC4964937; doi:10.1111/cod.12334)
Supplement: Supplementary file 1 — Appendix S1. Chromium released from leather – II: the importance of environmental parameters. [file COD-72-275-s001.docx]

**Supplementary information**

**Chromium released from leather – II: The importance of environmental parameters**

Frederik Mathiason, Carola Lidén, and Yolanda Hedberg

Table of content: Table S1, Figure S1.

***Table S1.*** *Chromium release from leather under different environmental conditions. Corresponding to the values described in Figs. 2-7 and S1, here in the unit mg/kg_leather_ including mean value and standard deviation. All results in Fig. 3-7 and S1 are from the same leather sample (cattle).*

| **Figure and condition** | | **Total Cr** | **Cr(III)** | **Cr(VI)** |
| --- | --- | --- | --- | --- |
|  |  | Mean (± SD) | Mean (± SD) | Mean (± SD) |
| **Fig. 2** | Cattle | 99.0 (23.8) | 91.1 (22.1) | 7.8 (2.0) |
|  | Pig | 143.7 (8.8) | 125.4 (8.4) | 18.2 (0.8) |
| **Figs. 3a and 3c** | RH 20 % (70 °C) | 99.0 (23.8) | 91.1 (22.1) | 7.8 (2.0) |
|  | RH 35 % (70 °C) | 85.1 (2.0) | 81.6 (3.5) | 3.5 (1.5) |
|  | RH 50 % (70 °C) | 59.3 (2.2) | 59.3 (2.2) | <LOD |
|  | RH 80 % (70 °C) | 22.3 (1.6) | 22.3 (1.6) | <LOD |
| **Fig. 3d** | RH 20 % (20 °C) | 123 (2.7) | 118 (1.6) | 5.0 (1.2) |
| **Figs. 4a and 4b** | First: pH 12 solution | 366.9 (29.4) | 357.5 (28.7) | 9.4 (2.9) |
|  | Second: PB | 131.4 (8.1) | 123.5 (7.6) | 7.9 (1.8) |
| **Figs. 5a and 5b** | PB after pH 12 without UV | 131.4 (8.1) | 123.5 (7.6) | 7.9 (1.8) |
|  | PB after pH 12 with UV | 143.0 (17.0) | 128.0 (15.2) | 15.0 (0.4) |
|  | PB without UV | 99.0 (23.8) | 95.5 (23.0) | 3.5 (1.5) |
|  | PB with UV | 92.0 (6.3) | 82.9 (5.7) | 9.0 (1.1) |
| **Fig. 6a** | First: rain 6 h | 414.1 (95.8) | 414.1 (95.8) | <LOD |
|  | Second: rain 6 h | 213.1 (38.0) | 213.1 (38.0) | <LOD |
|  | Third: rain 6 h | 139.7 (30.6) | 139.7 (30.6) | <LOD |
| **Figs. 6b and 6c** | First: 3 h rain | 285.5 (16.9) | 285.5 (16.9) | <LOD |
|  | Second: 3 h PB | 69.4 (19.1) | 58.9 (16.3) | 10.4 (3.7) |
|  | First: 6 h rain | 427.4 (32.1) | 427.4 (32.1) | <LOD |
|  | Second: 3 h PB | 74.0 (4.8) | 64.1 (4.2) | 9.8 (0.4) |
| **Figs. 7a and 7b** | Rain (pH 4.3) | 285.5 (16.9) | 285.5 (16.9) | <LOD |
|  | ASW (pH 6.5) | 312.6 (68.1) | 312.6 (68.1) | <LOD |
|  | PB (pH 8.0) | 99.0 (23.8) | 91.1 (21.9) | 7.8 (2.0) |
|  | Alkaline solution (pH 12) | 366.9 (29.4) | 357.5 (28.7) | 9.4 (2.9) |
| **Fig. S1** | Worn leather | 70.9 (13.3) | 67.2 (12.6) | 3.7 (3.0) |
|  | Intact leather | 99.0 (23.8) | 91.1 (22.1) | 7.8 (2.0) |

ASW, artificial sweat; LOD, limit of determination; PB, phosphate buffer; rain, artificial rain; RH, relative humidity; SD, standard deviation;


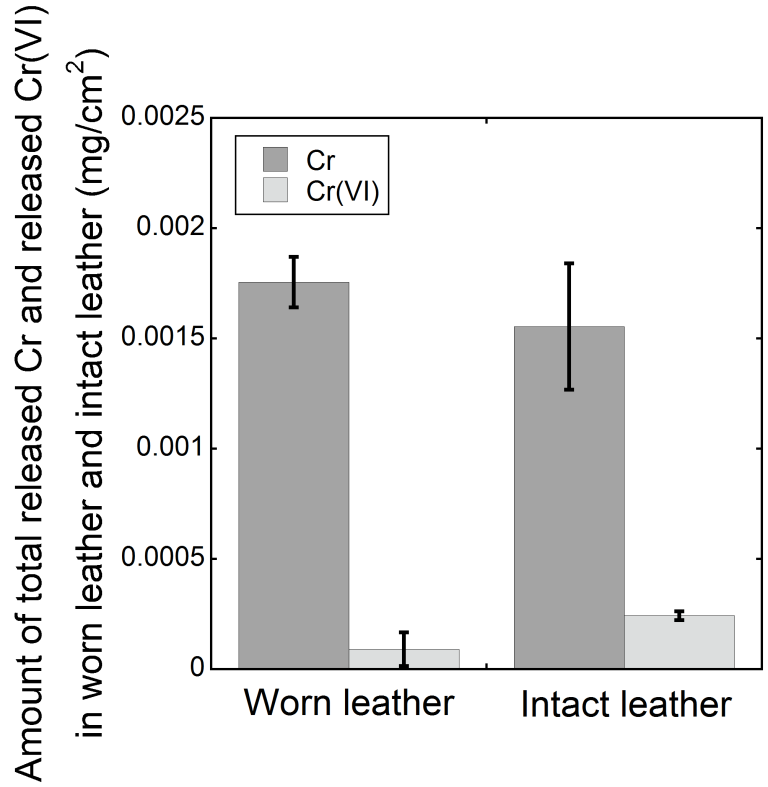


***Figure S1.*** *Released amounts of total Cr and Cr(VI) from artificially worn (stretched) leather and intact leather in phosphate buffer (PB) after 3 h of extraction at room temperature (20 – 25 °C). Prior to extraction, the leather was stored and conditioned at 70 °C, RH 20%, for 24 h. No significant differences were observed for the samples (Cr: p = 0.3499; Cr(VI): p = 0.0649). The error bars indicate standard deviation between triplicate samples.*
